# Plasma metabolomic profile in orthostatic intolerance children with high levels of plasma homocysteine

**DOI:** 10.1186/s13052-024-01601-4

**Published:** 2024-03-14

**Authors:** Yaqi Li, Baoling Bai, Hui Wang, Haojie Wu, Yanjun Deng, Chen Shen, Qin Zhang, Lin Shi

**Affiliations:** 1 Department of Cardiology, Children’s Hospital, Capital Institute of Pediatrics, No 2 Yabao Road, Beijing, Chaoyang District 100020 China; 2 Beijing Municipal Key Laboratory of Child Development and Nutriomics, Capital Institute of Pediatrics, No 2 Yabao Road, Beijing, Chaoyang District 100020 China

**Keywords:** Metabolomics, Homocysteine, Orthostatic intolerance, Children

## Abstract

**Background:**

Orthostatic intolerance, which includes vasovagal syncope and postural orthostatic tachycardia syndrome, is common in children and adolescents. Elevated plasma homocysteine levels might participate in the pathogenesis of orthostatic intolerance. This study was designed to analyze the plasma metabolomic profile in orthostatic intolerance children with high levels of plasma homocysteine.

**Methods:**

Plasma samples from 34 orthostatic intolerance children with a plasma homocysteine concentration > 9 µmol/L and 10 healthy children were subjected to ultra-high-pressure liquid chromatography and quadrupole-time-of-flight mass spectrometry analysis.

**Results:**

A total of 875 metabolites were identified, 105 of which were significantly differential metabolites. Choline, 1-stearoyl-2-linoleoyl-*sn*-glycero-3-phosphocholine, 1-(1*Z*-octadecenyl)-2-(4*Z*,7*Z*,10*Z*,13*Z*,16*Z*,19*Z*-docosahexaenoyl)-*sn*-glycero-3-phosphocholine, histidine, isocitric acid, and DL-glutamic acid and its downstream metabolites were upregulated, whereas 1-palmitoyl-*sn*-glycero-3-phosphocholine, 1-stearoyl-*sn*-glycerol 3-phosphocholine, sphingomyelin (d18:1/18:0), betaine aldehyde, hydroxyproline, and gamma-aminobutyric acid were downregulated in the orthostatic intolerance group compared with the control group. All these metabolites were related to choline and glutamate. Heatmap analysis demonstrated a common metabolic pattern of higher choline, 1-stearoyl-2-linoleoyl-*sn*-glycero-3-phosphocholine, and DL-glutamic acid, and lower sphingomyelin (d18:1/18:0), 1-stearoyl-*sn*-glycerol 3-phosphocholine, and 1-palmitoyl-*sn*-glycero-3-phosphocholine in patients with certain notable metabolic changes (the special group) than in the other patients (the common group). The maximum upright heart rate, the change in heart rate from the supine to the upright position, and the rate of change in heart rate from the supine to the upright position of vasovagal syncope patients were significantly higher in the special group than in the common group (*P* < 0.05). Choline, 1-stearoyl-2-linoleoyl-*sn*-glycero-3-phosphocholine, and DL-glutamic acid were positively correlated with the rate of change in heart rate from the supine to the upright position in vasovagal syncope patients (*P* < 0.05).

**Conclusions:**

The levels of choline-related metabolites and glutamate–related metabolites changed significantly in orthostatic intolerance children with high levels of plasma homocysteine, and these changes were associated with the severity of illness. These results provided new light on the pathogenesis of orthostatic intolerance.

**Supplementary Information:**

The online version contains supplementary material available at 10.1186/s13052-024-01601-4.

## Background

Orthostatic intolerance (OI) refers to the inability to tolerate the upright position due to several symptoms (e.g., dizziness, headache, palpitation, and even syncope), which can be relieved by lying down [1]. Vasovagal syncope (VVS) and postural orthostatic tachycardia syndrome (POTS) are the two main forms of OI, accounting for approximately 75% of children with syncope [2, 3]. OI children experience serious physical and mental health issues that lower their quality of life. However, the pathogenesis of OI is complex and has not been completely understood. Previously, we reported that the plasma homocysteine (Hcy) levels in POTS children (9.78 [7.68, 15.31] µmol/L) were significantly higher than those in control children (7.79 [7.46, 9.63] µmol/L) (*P* < 0.05), and that Hcy levels were positively correlated with the change in heart rate from the supine to the upright position (ΔHR) and the rate of change in heart rate from the supine to the upright position (ΔHR/sHR × 100%) during the head-up tilt test (HUTT) [4]. Children with VVS also had significantly higher serum Hcy levels than control children (13.55 ± 5.03 vs. 7.81 ± 1.71 µmol/L, *P* < 0.05), and Hcy levels were positively correlated with the average PQ interval and average QTc interval during 24-h Holter monitoring [5]. The above indices are important to reflect the severity of OI. Therefore, these studies indicated that high levels of plasma Hcy might participate in the pathogenesis of OI.

Hcy can cause a series of negative effects, including promotion of oxidative stress and inflammation, autonomic nerve dysfunction, and injury to the vascular endothelium [6–9]. Plasma Hcy levels in children are lower than those in adults [10]. Taking after earlier studies [4, 5], this study defined a high plasma Hcy concentration as > 9 µmol/L. However, it remains unknown how the plasma metabolites change in OI children with high levels of plasma Hcy and whether these changes are related to the pathogenesis of OI. To address these questions, we used ultra-high-pressure liquid chromatography and quadrupole-time-of-flight mass spectrometry (UHPLC-Q-TOF MS) to investigate the differences in metabolites between the plasma samples from OI children with high levels of plasma Hcy and those from healthy children. The results will enhance our understanding of the pathogenesis of OI at the molecular level.

## Methods

### Study participants

The OI group included 34 OI children with high levels of plasma Hcy (23 with VVS and 11 with POTS and plasma Hcy > 9 µmol/L), who were admitted to our hospital from February 2021 to October 2021 due to OI symptoms. All children fulfilled the following diagnostic criteria for VVS or POTS: (1) mainly older children; (2) presence of OI symptoms; (3) positive HUTT; and (4) absence of other conditions that might cause similar symptoms, such as cerebrovascular disorders and organic heart diseases [2]. Ten children without previous syncopal or presyncopal episodes were enrolled in the control group. All individuals’ physical examination and biochemistry findings were normal. They were also free of any history of chronic organic conditions, such as hypothyroidism, hepatic disorders, or kidney diseases. The general data of all subjects were collected, including gender, age, body mass index (BMI), mean supine heart rate (sHR), mean supine systolic blood pressure (sSBP), and mean supine diastolic blood pressure (sDBP).

### Head-up tilt test

The protocols for the basic HUTT (BHUTT) and the sublingual nitroglycerin-provoked HUTT (SNHUTT) were in accordance with the previously published guideline [2]. BHUTT: Drugs affecting the autonomic nervous system’s function were discontinued 3 days before the test. After 12 h of fasting, the children were tested in a quiet, dark room. The blood pressure (BP), heart rate (HR), and electrocardiogram (ECG) results were recorded after the child had been lying on the tilt table at least 10 min, and then the table was adjusted to lean at a 60-degree angle for 45 min while the children’s BP, HR, and ECG were simultaneously measured. Once a positive reaction or OI symptoms appeared, the child was returned to the supine position. SNHUTT: Children underwent SNHUTT if syncope or presyncope did not occur with the BHUTT. Under the simultaneous recording of BP, HR, ECG, and clinical performance, they took 4–6 µg/kg sublingual nitroglycerin and remained tilted at 60-degree angle for an additional 20 min. The endpoint was a positive response or completion of the process. The positive response during the HUTT for VVS or POTS was defined according to the previous guideline [2]. There are three subtypes of VVS according to the patient’s response, including VVS vasoinhibitory type (VVS-VI), VVS cardioinhibitory type (VVS-CI), and VVS mixed type (VVS-M).

The ΔHR was calculated as the maximum upright HR (max-uHR) minus the mean supine HR (sHR). The value of ΔHR/sHR × 100% was calculated by dividing ΔHR by the sHR and multiplying by 100. For VVS, the change in SBP (ΔSBP) was also calculated as the minimum upright SBP (min-uSBP) minus the mean supine SBP (sSBP), and the rate of change in SBP (ΔSBP/sSBP × 100%) was determined. Similarly, the change in DBP (ΔDBP) and the rate of change in DBP (ΔDBP/sDBP × 100%) of VVS were calculated. These hemodynamic indices objectively reflect the severity of POTS or VVS.

### Plasma sample collection and preparation

Peripheral blood was drawn from the participants and collected using anticoagulated tubes after 8–12 h of fasting. Then, the plasma was separated and kept at − 80 °C. To clear the protein, 100 µL of plasma was mixed with 400 µL of methanol/acetonitrile (1:1, v/v) and was centrifuged at 14,000×*g* for 15 min at 4 °C. Then, the supernatant was dried in a vacuum. Finally, the samples were treated in acetonitrile/water (1:1, v/v) solvent. The same process was used to prepare the quality-control samples.

### UHPLC-Q-TOF MS analysis

Analysis was performed by an ultra-high-pressure liquid chromatography (1290 Infinity LC, Agilent Technologies) coupled to a quadrupole time-of-flight (Triple TOF 6600, AB SCIEX). For hydrophilic interaction liquid chromatography separation, a 2.1 mm × 100 mm ACQUITY UPLC BEH 1.7 μm column (Waters, Ireland) was used. The mobile phase contained A = 25 mM of ammonium acetate and 25 mM of ammonium hydroxide in water and B = acetonitrile. The gradient was: 95% B (0-0.5 min), 95–65% B (0.5-7 min), 65–40% B (7–8 min), 40% B (8–9 min), 40–95% (9-9.1 min), and 95% B (9.1–12 min).

After separation, the samples were detected by Triple TOF 6600 through electrospray ionization (ESI) positive and negative modes. The apparatus was set up for MS-only collection with a *m/z* range of 60–1,000 Da and a TOF MS scan accumulation time of 0.20 s/spectrum. For automatic MS/MS collection, a *m/z* range of 25–1,000 Da and a product ion scan accumulation time of 0.05 s/spectra were used. Information-dependent acquisition was applied to gather the product ion scan in the selected high-sensitivity mode with the following conditions: the collision energy = 35 ± 15 eV and declustering potential = ± 60 V. Isotopes within 4 Da were excluded. Ten candidate ions were monitored during each cycle.

### Data analysis

Gender (male/female ratio) and VVS subtype (VVS-VI/CI/M ratio) were compared between the two groups by the chi-square test or Fisher’s exact test. Age, BMI, and hemodynamic indices were compared by the independent Student’s *t* test. Plasma Hcy levels were analyzed by the Mann–Whitney *U* test. *P <* 0.05 was considered statistically significant.

Metabolites were identified by comparing the accuracy of the *m*/*z* values (< 10 ppm) and the MS/MS spectra of various databases, including the Kyoto Encyclopedia of Genes and Genomes (KEGG) and an in-house database. Following sum normalization, the processed data were subjected to multivariate data analysis, which included Pareto-scaled principal component analysis (PCA) and orthogonal partial least-squares discriminant analysis (OPLS-DA). In the OPLS-DA model, each variable’s contribution to the classification was evaluated by the variable importance in projection (VIP) value. The significance of differences between two groups of independent samples was assessed by Student’s *t* test. The fold change (FC) > 1.5 and *P* < 0.05 indicated upregulation. The FC < 0.67 and *P* < 0.05 indicated downregulation. The criteria for identifying significantly different metabolites were VIP > 1 and *P* < 0.05.

Then, KEGG analysis was used to examine the interactions of metabolites in the metabolic pathways. Fisher ‘s exact test was used to calculate the significance level of metabolite enrichment in each pathway. The pathway significance level was higher when the *P* value was lower.

To further identify similar metabolic profiles among the samples, heatmaps were generated through hierarchical clustering analysis (HCA). The average linkage clustering approach and Euclidian distance were used to perform HCA of the samples in the heatmap. To investigate the potential relationships between metabolites, a correlation-based network analysis was performed by calculating Spearman’s correlation coefficients. Cytoscape was used to visualize the correlation-based network [11]. Only correlations with |r| > 0.8 and *P* < 0.05 were presented.

Finally, Spearman’s correlation analysis was used to assess the associations between metabolites and clinical indices. *P <* 0.05 was considered statistically significant.

## Results

### Characteristics of the participants

To identify the related plasma metabolites in OI children with high levels of plasma Hcy, plasma samples from 34 OI children with plasma Hcy > 9 µmol/L and 10 healthy controls were collected. The relevant information of all participants is shown in Table 1. The plasma Hcy levels of OI group were significantly higher than those of control group (11.33 [10.00, 13.64] vs. 8.12 [7.62, 9.02] µmol/L (*P <* 0.05; Table 1). No other characteristics were significantly different.

**Table 1:** Characteristics of the participants

Characteristics	OI group	Control group	χ^2^/t/Z	**P** value
Cases, n	34	10	-	-
Males/females, n	19/15	5/5	0.728	0.473^a^
Age, years	12.91 ± 1.83	12.00 ± 2.54	1.264	0.213^b^
BMI, kg/m^2^	20.18 ± 4.30	23.33 ± 5.77	−1.880	0.067^b^
sHR, bpm	74.73 ± 12.67	80.00 ± 10.10	−1.030	0.310^b^
sSBP, mmHg	109.29 ± 10.14	108.78 ± 13.59	0.126	0.900^b^
sDBP, mmHg	67.79 ± 7.85	67.22 ± 7.35	0.197	0.845^b^
Plasma Hcy, µmol/L	11.33 (10.00, 13.64)	8.12 (7.62, 9.02)	−4.593	0.000^c, *^

Values represent n, mean SD, or median (interquartile range). BMI, body mass index; sHR, mean supine heart rate; sSBP, mean supine systolic blood pressure; sDBP, mean supine diastolic blood pressure; Hcy, homocysteine; OI, orthostatic intolerance; a, chi-square test; b, independent-samples Student’s *t* test; c, Mann–Whitney *U* test; ^*^P < 0.05 vs. the control group

### Differential metabolites between the OI group and the control group

**Fig. 1:**
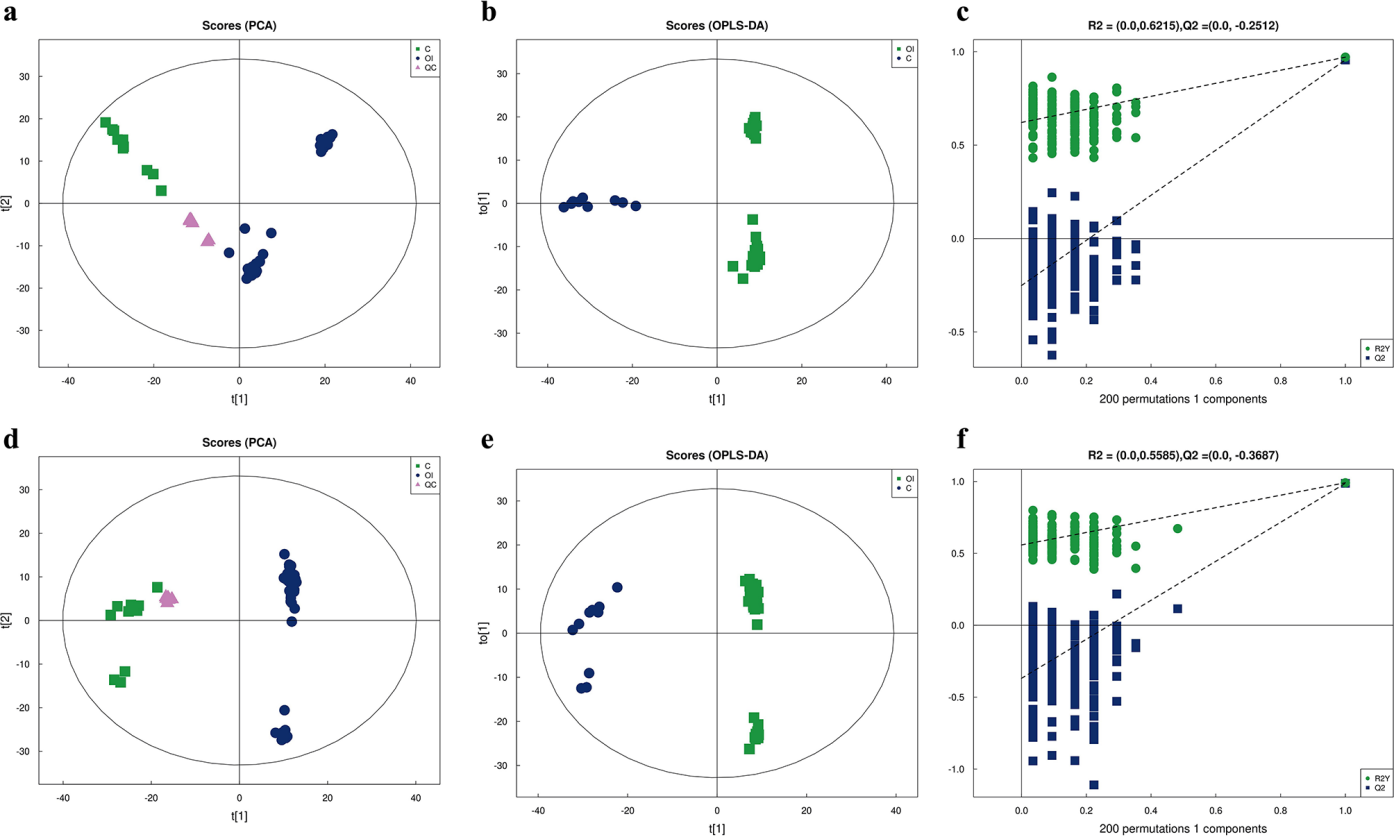
Chemometric analysis of metabolites. (**a**, **d**) PCA score plot of the positive ion mode and negative ion mode. (**b**, **e**) OPLS-DA score plot of the positive ion mode and negative ion mode. The samples’ score on the first principal component is shown by the x-axis, while their score on the second principal component is represented by the y-axis. The ellipse represents 95% confidence interval. Green points represent each biological repetition in the control group, and blue points represents each biological repetition in the OI group. Purple points represent QC samples. (**c**, **f**) The permutation test of OPLS-DA models. The x-axis stands for the displacement retention, and the y-axis stands for the values of R2 and Q2. Green points represent R2Y, blue points represent Q2, and two dotted lines represent the regression lines of R2 and Q2. In the top right corner, R2 and Q2 stand for permutation retention = 1. C: Control group. OI: OI group

Then, the plasma samples were subjected to UHPLC-Q-TOF MS. The processed data were analyzed by multivariate data analysis including PCA and OPLS-DA. Figure 1a and d show the PCA results of the overall samples. The quality control samples were closely clustered, demonstrating excellent instrument stability. The samples from the two groups were well distinguished by OPLS-DA (Fig. 1b and e), indicating a significant difference in the spectrum of plasma metabolism between the two groups. The model evaluation parameters, R2Y (cum) and Q2 (cum) were obtained by seven cycles of interaction verification. The model was stable and reliable, as positive ion mode Q2 (cum) = 0.916 and negative ion mode Q2 (cum) = 0.989. The validity of the OPLS-DA models was checked using the permutation test (Fig. 1c and f). The R2 and Q2 of the random model gradually fell as the permutation retention did, revealing that the original model was robust and not overfit.

**Fig. 2:**
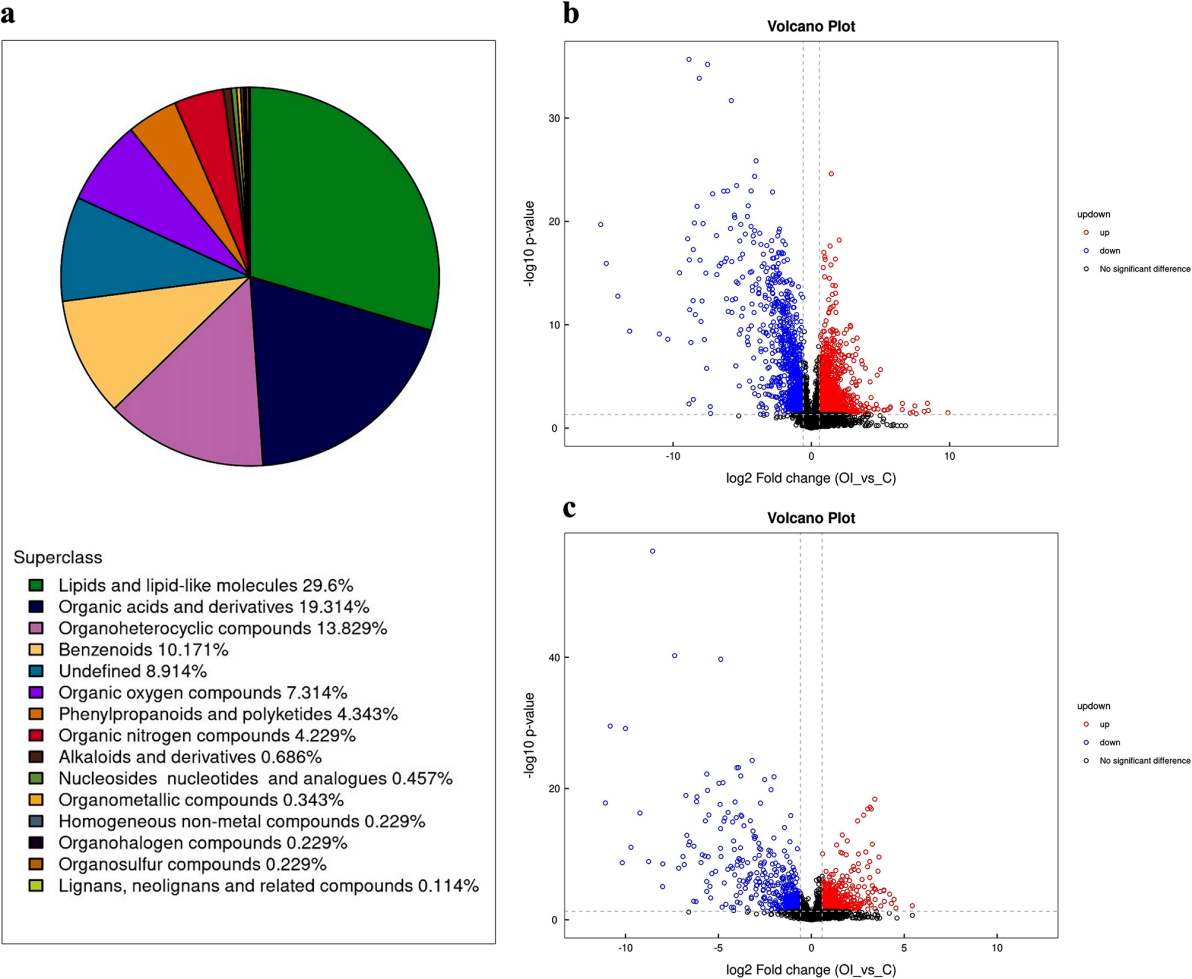
Metabolite classification analysis and volcano plots. (**a**) The pie chart shows 14 superclasses. (**b**, **c**) Volcano plots of positive ion mode and negative ion mode. Red points represent upregulated metabolites satisfying FC > 1.5 and *P* < 0.05, blue points represent downregulated metabolites satisfying FC < 0.67 and *P* < 0.05, and black points represent nondifferential metabolites. C: Control group. OI: OI group

A total of 875 metabolites were identified, belonging to 14 superclasses (Fig. 2a). To screen for potentially different metabolites, volcano plots were created based on the results of FC analysis and Student’s *t* test (Fig. 2b and c). Subsequently, we identified 105 significantly different metabolites with VIP > 1 and *P* < 0.05 (Supplementary Table S1). They mainly belonged to lipids and lipid-like molecules (24.8%), organic acids and derivatives (20.0%), benzenoids (15.2%), organoheterocyclic compounds (12.4%), and organic oxygen compounds (11.4%).

### KEGG metabolic pathway analysis of the differential metabolites

**Fig. 3:**
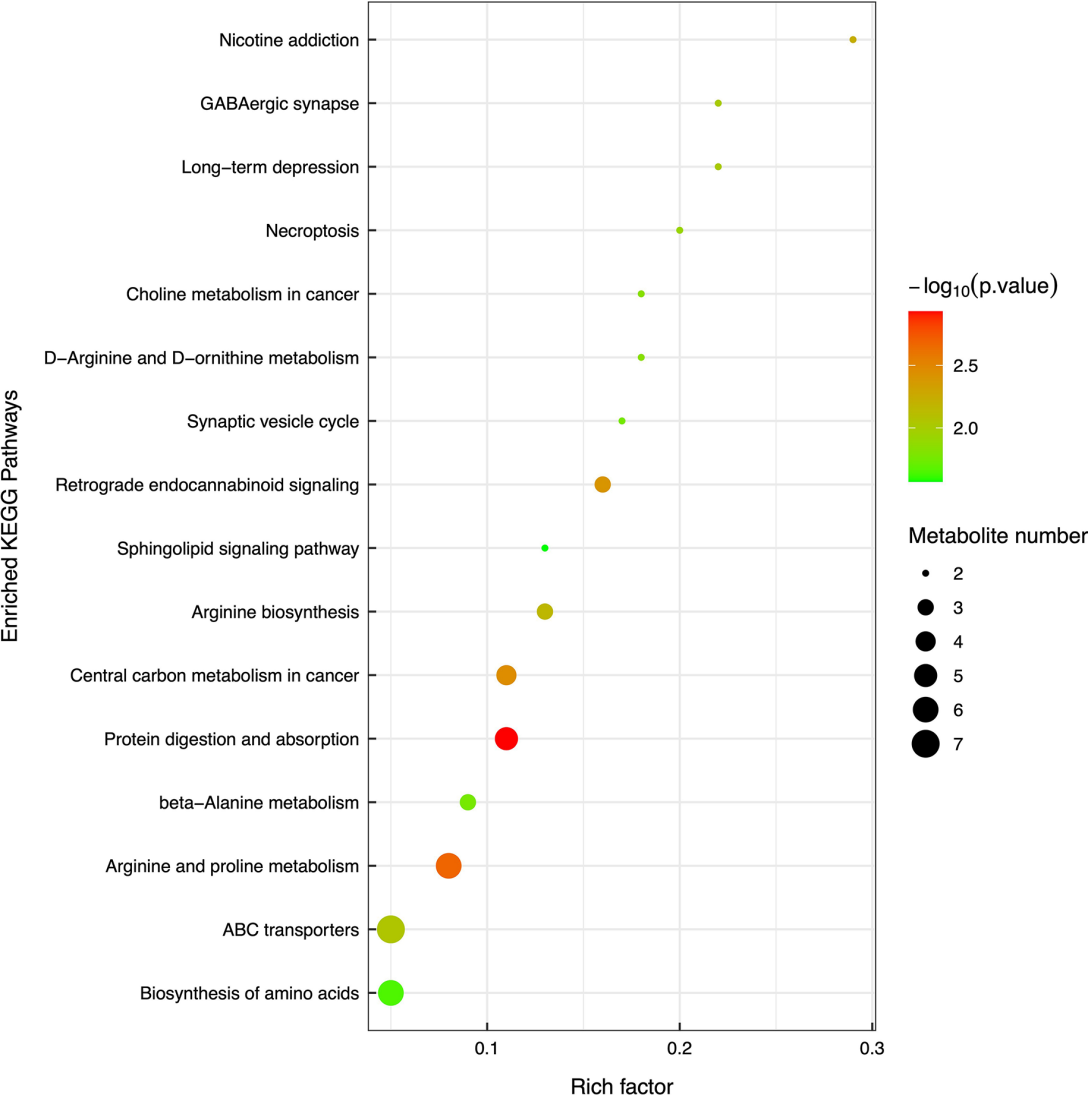
KEGG enrichment pathway diagram. Bubble size represents the influence factor of the pathway. It increases with the influence factor. Bubble color represents the negative common logarithm of *P* value (− log10 *P* value). The color becomes deeper as the *P* value descends, indicating higher pathway significance level. Rich factor represents the ratio of the number of differential metabolites annotated in the pathway

The metabolites were analyzed and included in the KEGG database to identify potential chemical and metabolic pathways that may be involved in OI with high levels of Hcy. Figure 3 displays the top 20 pathways with the highest significance level according to *P* value.

### Choline-related metabolites and glutamate-related metabolites

Among the 105 significantly differential metabolites, 7 choline-related metabolites and 9 glutamate-related metabolites, which might be associated with OI according to previous studies, were chosen for further study. The identification results are shown in Tables 2 and 3. Among the choline-related metabolites, 1-(1*Z*-octadecenyl)-2-(4*Z*,7*Z*,10*Z*,13*Z*,16*Z*,19*Z*-docosahexaenoyl)-*sn*-glycero-3-phosphocholine [PC(P-18:0/22:6(4*Z*,7*Z*,10*Z*,13*Z*,16*Z*,19*Z*))], choline, and 1-stearoyl-2-linoleoyl-*sn*-glycero-3-phosphocholine [PC(18:0/18:2(9*Z*,12*Z*))] were upregulated, whereas 1-palmitoyl-*sn*-glycero-3-phosphocholine [LPC(16:0)], 1-stearoyl-*sn*-glycerol 3-phosphocholine [LPC(18:0)], sphingomyelin (d18:1/18:0), and betaine aldehyde were downregulated. Among the glutamate-related metabolites, 4-hydroxy-l-glutamic acid, *N*-alpha-acetyl-l-ornithine, DL-glutamic acid, isocitric acid, DL-proline, histidine, and ornithine were upregulated, whereas hydroxyproline and gamma-aminobutyric acid (GABA) were downregulated. The above metabolites were mainly involved in the arginine and proline metabolism and in the biosynthesis of amino acids (the KEGG pathway map is shown in Supplementary Figures S1 and S2). The differential metabolism of choline and DL-glutamic acid between the two groups indicated that they might play a role in OI children with high Hcy levels.

**Table 2:** Choline-related metabolites

Adduct	Description	VIP	FC	*P* value	m/z	rt s
[M + H]+	PC(P-18:0/22:6 (4*Z*,7*Z*,10*Z*,13*Z*,16*Z*,19*Z*))	1.12864324	32.04534983	2.2299E-06	818.6056	270.3795
[M]+	Choline	9.57410073	22.7689174	0.01430261	104.10746	385.594
[M + H]+	PC(18:0/18:2(9*Z*,12*Z*))	12.4282163	4.100921334	0.02764031	786.60081	265.873
[M + H]+	LPC (16:0)	11.7194728	0.448253038	0.0001294	496.33986	284.074
[M + H]+	LPC (18:0)	7.3755837	0.443874234	0.00027298	524.37111	281.046
[M + H]+	Sphingomyelin (d18:1/18:0)	2.06610853	0.35067383	8.1599E-08	731.60532	273.912
[M + H]+	Betaine aldehyde	1.87630121	0.01239109	1.1838E-23	103.08705	102.509

VIP, variable importance in projection; FC, fold change; PC(P-18:0/22:6(4*Z*,7*Z*,10*Z*,13*Z*,16*Z*,19*Z*)), 1-(1*Z*-octadecenyl)-2-(4*Z*,7*Z*,10*Z*,13*Z*,16*Z*,19*Z*-docosahexaenoyl)-*sn*-glycero-3-phosphocholine; PC(18:0/18:2(9*Z*,12*Z*)), 1-stearoyl-2-linoleoyl-*sn*-glycero-3-phosphocholine; LPC (16:0), 1-palmitoyl-*sn*-glycero-3-phosphocholine; LPC (18:0), 1-stearoyl-*sn*-glycerol 3-phosphocholine

**Table 3:** Glutamate-related metabolites

Adduct	Description	VIP	FC	P value	m/z	rt s
[M + H-H2O]+	4-hydroxy-l- glutamic acid	1.0174839	7.89610076	0.00092132	146.02716	375.068
[M + H-H2O]+	N-.alpha.-acetyl-l-ornithine	1.36799865	6.59685522	0.00016345	157.09737	413.397
[M + H]+	DL-glutamic acid	1.60444528	3.21295374	0.04575234	148.04265	412.758
[M-H-H2O]-	Isocitric acid	4.78307938	2.69163527	0.01590389	172.99091	36.45055
[M + H]+	DL-proline	2.67987819	2.59975309	0.00565456	116.071	589.936
[M + H]+	Histidine	5.12808658	1.88791825	0.00087118	156.07683	463.903
[M + H]+	Ornithine	1.02559587	1.82911683	0.00178978	133.09828	616.07
[M + H]+	Hydroxyproline	1.24142253	0.05095732	9.8938E-13	132.08096	80.1036
[M + H-H2O]+	GABA	2.65109886	0.03649299	1.6886E-19	86.06064	85.9618

VIP, variable importance in projection; FC, fold change; GABA, gamma-aminobutyric acid

### Hierarchical clustering analysis and correlation-based network analysis of differential metabolites

**Fig. 4:**
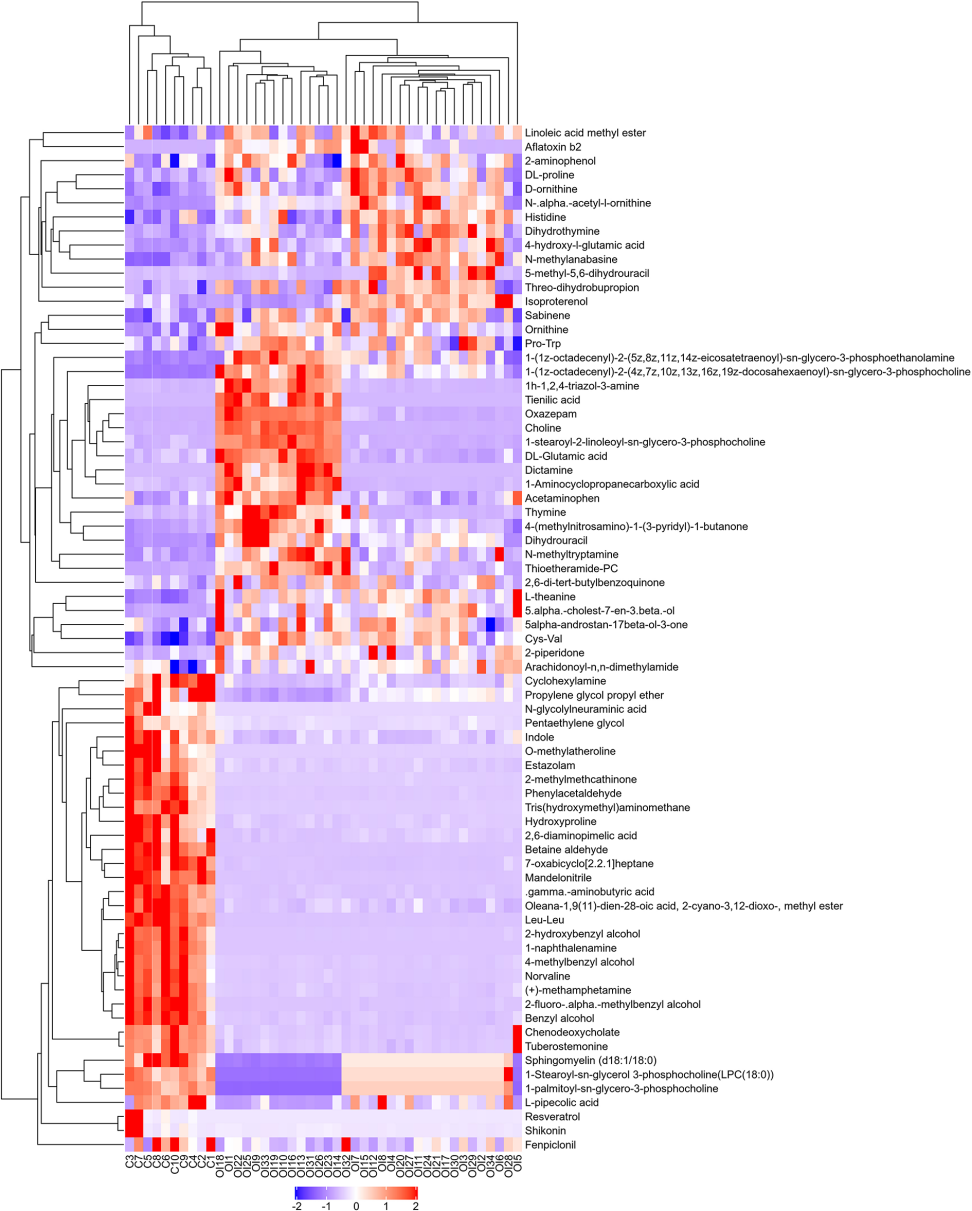
Heatmap of positive ion mode. Each row in the y-axis represents a significantly differential metabolite and each column in the x-axis represents a set of samples. Red represents significant upregulation of the metabolite, and blue represents significant downregulation of the metabolite. The degree of upregulation and downregulation is represented by the color depth. The metabolites with similar expression patterns are clustered in the same cluster on the left. C: Control group. OI: OI group

To provide intuitive visualization of the metabolic differences between OI patients and controls, we created heatmaps through HCA. Figure 4 shows the heatmap of the results from positive ion mode and Supplementary Figure S3 shows the heatmap of the results from negative ion mode, demonstrating clear differential metabolic patterns between the two groups.

**Fig. 5:**
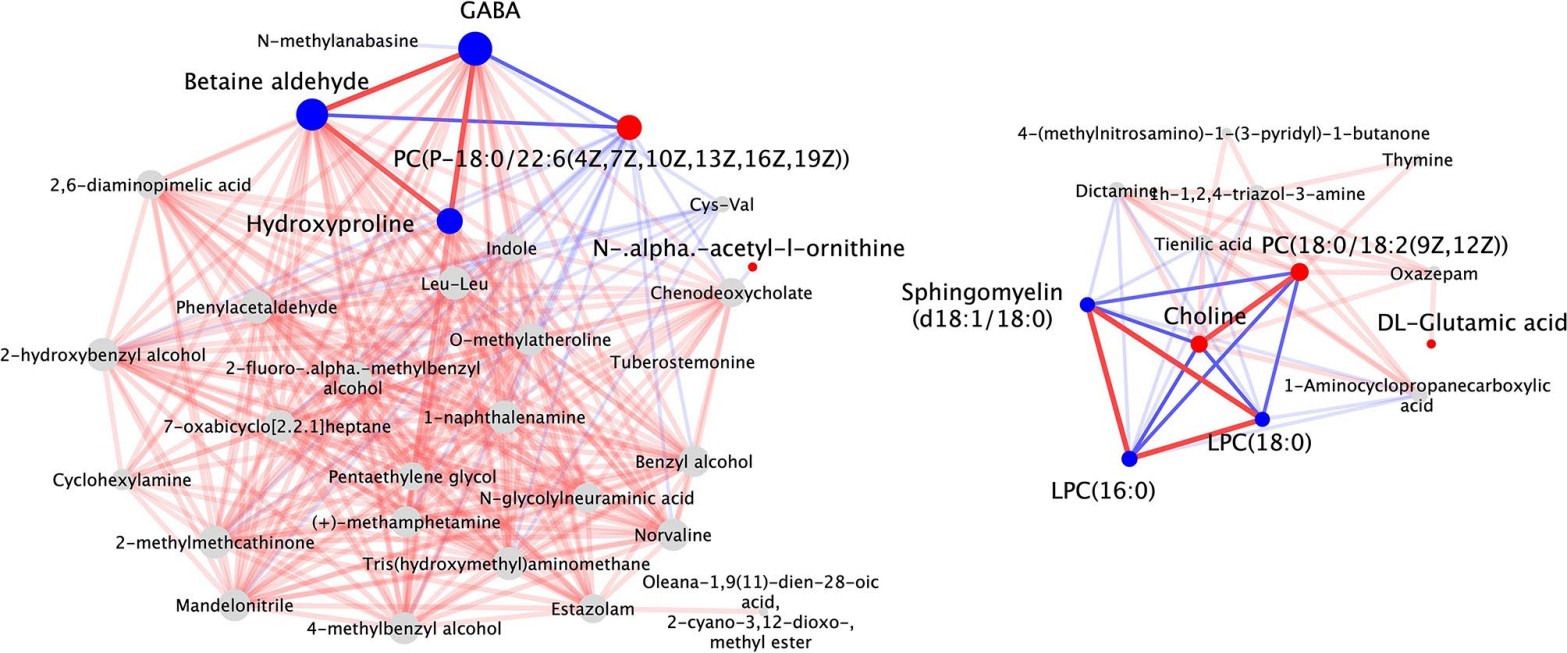
Correlation-based network of positive ion mode. Each point represents a significantly differential metabolite. Red points and blue points are upregulated and downregulated metabolites of interest, respectively. The sizes of the points increase with degree. The positive and negative relationships between metabolites with Spearman correlation coefficient|r| > 0.8 and *P* < 0.05 are shown in red and blue lines respectively. The solid lines represent the correlations of interest. The thickness of lines represents absolute correlation coefficient. The line becomes thicker as the absolute correlation coefficient increases. GABA, gamma-aminobutyric acid; PC(P-18:0/22:6(4*Z*,7*Z*,10*Z*,13*Z*,16*Z*,19*Z*)), 1-(1*Z*-octadecenyl)-2-(4*Z*,7*Z*,10*Z*,13*Z*,16*Z*,19*Z*-docosahexaenoyl)-*sn*-glycero-3-phosphocholine; PC(18:0/18:2(9*Z*,12*Z*)), 1-stearoyl-2-linoleoyl-*sn*-glycero-3-phosphocholine; LPC (16:0), 1-palmitoyl-*sn*-glycero-3-phosphocholine; LPC (18:0), 1-stearoyl-*sn*-glycerol 3-phosphocholine

Next, to illustrate the relationship between two significantly differential metabolites, we drew correlation-based networks. Figure 5 shows the network of the results from positive ion mode and Supplementary Figure S4 shows the network of the results from negative ion mode. GABA and betaine aldehyde were positively correlated with hydroxyproline and negatively correlated with PC(P-18:0/22:6(4*Z*,7*Z*,10*Z*,13*Z*,16*Z*,19*Z*)). Choline was positively correlated with PC(18:0/18:2(9*Z*,12*Z*)) and negatively correlated with LPC(16:0), LPC(18:0), and sphingomyelin (d18:1/18:0).

### Relationships between metabolic changes and clinical indices

**Fig. 6:**
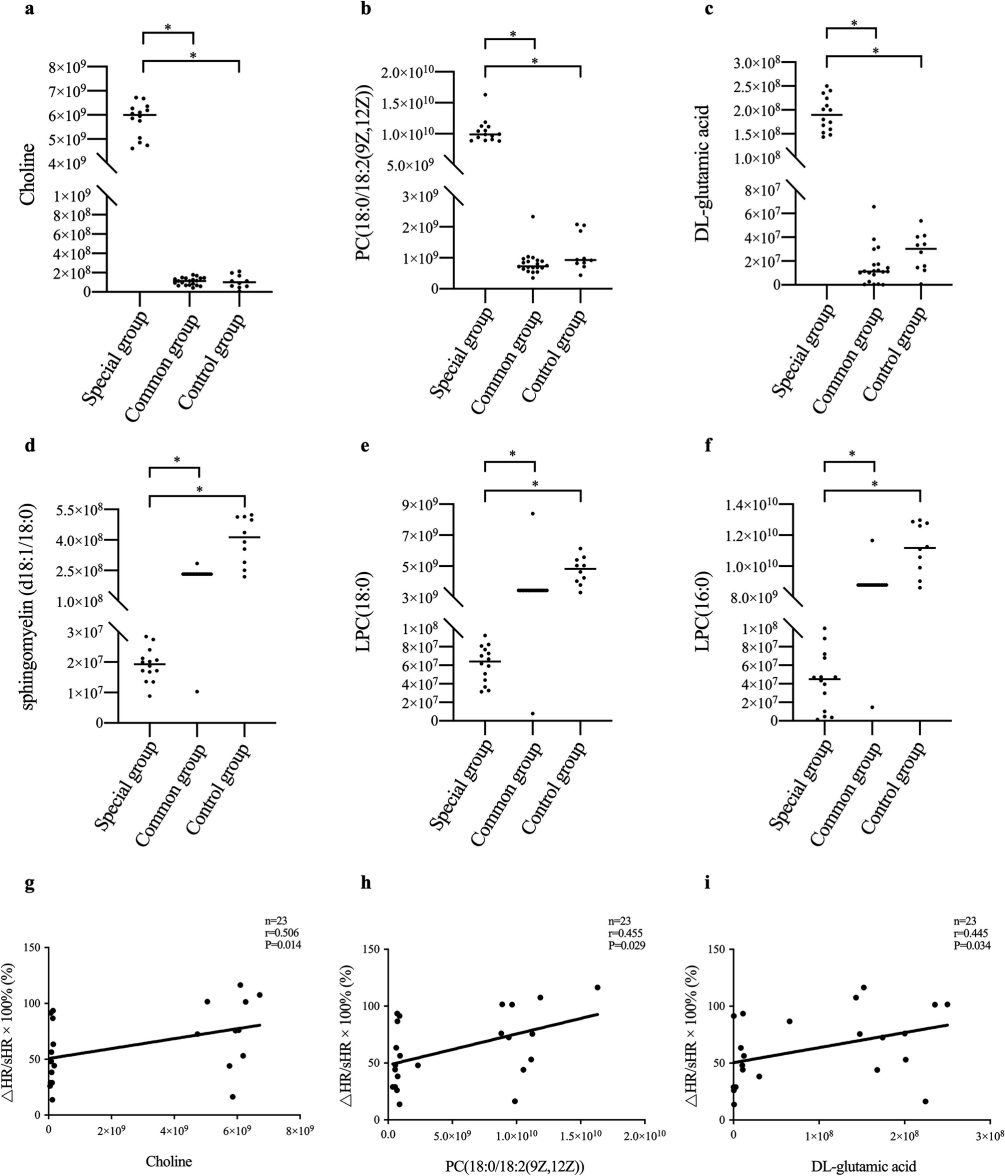
Relationships between metabolic changes and clinical indices. (**a**-**f**) Comparison of differential metabolites among the three groups. ^*^P < 0.05 vs. the common group and the control group. (**g**-**i**) Relationship between metabolites and △HR/sHR × 100% in VVS patients. △HR/sHR × 100%, rate of change in heart rate from the supine to the upright position; PC(18:0/18:2(9*Z*,12*Z*)), 1-stearoyl-2-linoleoyl-*sn*-glycero-3-phosphocholine

By analyzing the heatmaps from positive ion mode, we also found that there were 14 OI patients exhibited notable metabolic changes (the special group). Compared with other patients (the common group) and healthy controls, these patients had higher plasma levels of choline, PC(18:0/18:2(9*Z*,12*Z*)), and DL-glutamic acid and lower plasma levels of sphingomyelin (d18:1/18:0), LPC(18:0), and LPC(16:0) (Fig. 6a-f).

To further investigate whether metabolic changes affected the severity of OI, we analyzed the relationships between metabolic changes and clinical indices. In the special group of OI, there were 10 VVS children and 4 POTS children. In the common group, there were 13 VVS children and 7 POTS children. Therefore, we compared VVS patients and POTS patients of these two groups separately. Gender, age, BMI, and plasma Hcy levels did not significantly differ between the two groups of VVS patients (*P* > 0.05; Table 4). However, VVS patients in the special group had higher max-uHR, ΔHR, and ΔHR/sHR × 100% during the HUTT than those in the common group (*P <* 0.05; Table 5). Moreover, choline, PC(18:0/18:2(9*Z*,12*Z*)), and DL-glutamic acid were positively correlated with ΔHR/sHR × 100% in VVS patients (*P <* 0.05; Fig. 6g-i), which indicated that metabolic changes were associated with the severity of VVS. However, no significant differences or correlations were found in POTS patients (*P* > 0.05; Supplementary Table S2 and Figure S5).

**Table 4:** Baseline characteristics and plasma Hcy levels of VVS patients

Characteristics	Special group	Common group	χ^2^/t/Z	*P* value
Cases, n	10	13	-	-
Males/females, n	4/6	7/6	-	0.680^a^
Age, years	13.08 ± 2.33	12.70 ± 1.83	0.421	0.678^b^
BMI, kg/m^2^	21.89 ± 4.72	18.68 ± 4.33	1.672	1.109^b^
Plasma Hcy, µmol/L	10.95 (9.94, 13.06)	11.17 (9.80, 15.07)	−0.279	0.780^c^

Values represent n, mean SD, or median (interquartile range). BMI, body mass index; Hcy, homocysteine; VVS, vasovagal syncope; a, Fisher’s exact test; b, independent-samples Student’s *t* test; c, Mann–Whitney *U* test

**Table 5:** Hemodynamic indices of VVS patients in the HUTT

Characteristics	Special group	Common group	χ^2^/t	*P* value
Cases, n	10	13	-	-
VVS-VI/CI/M, n	7/0/3	8/1/4	0.832	0.660^a^
sHR, bpm	73.20 ± 15.17	73.85 ± 12.29	−0.113	0.911^b^
max-uHR, bpm	125.40 ± 11.10	110.46 ± 19.78	2.136	0.045^b, *^
△HR, bpm	52.20 ± 17.29	36.62 ± 17.94	2.098	0.048^b, *^
△HR/sHR × 100%, %	76.49 ± 31.71	51.36 ± 26.02	2.089	0.049^b, *^
sSBP, mmHg	111.30 ± 12.00	113.46 ± 7.21	−0.538	0.597^b^
min-uSBP, mmHg	85.60 ± 10.58	91.08 ± 5.58	−1.607	0.123^b^
△SBP, mmHg	−25.70 ± 13.28	−22.38 ± 9.01	−0.713	0.483^b^
△SBP/sSBP × 100%, %	−22.56 ± 10.73	−19.43 ± 7.10	−0.842	0.409^b^
sDBP, mmHg	63.10 ± 4.84	64.85 ± 6.15	−0.738	0.469^b^
min-uDBP, mmHg	39.70 ± 8.37	43.92 ± 9.26	−1.130	0.271^b^
△DBP, mmHg	−23.40 ± 10.44	−20.92 ± 10.02	−0.577	0.570^b^
△DBP/sDBP × 100%, %	−36.58 ± 15.44	−31.90 ± 14.36	−0.750	0.462^b^

Values represent n, mean SD, or median (interquartile range). HUTT, head-up tilt test; VVS, vasovagal syncope; VI, vasoinhibitory type; CI cardioinhibitory type; M mixed type; sHR, mean supine heart rate; max-uHR, maximum upright heart rate; △HR, change in heart rate from the supine to the upright position (max-uHR– sHR); △HR/sHR × 100%, rate of change in heart rate from the supine to the upright position; sSBP, mean supine systolic blood pressure; min-uSBP, minimum upright systolic blood pressure; △SBP, change in systolic blood pressure from the supine to the upright position (min-uSBP– sSBP); △SBP/sSBP × 100%, rate of change in systolic blood pressure from the supine to the upright position; sDBP, mean supine diastolic blood pressure; min-uDBP, minimum upright diastolic blood pressure; △DBP, change in diastolic blood pressure from the supine to the upright position (min-uDBP– sDBP); △DBP/sDBP × 100%, rate of change in diastolic blood pressure from the supine to the upright position; a, chi-square test; b, independent-samples Student’s *t* test; ^*^P < 0.05 vs. the common group

## Discussion

OI is a prevalent functional cardiovascular disease that affects children and adolescents [1–3]. A series of hemodynamic changes, such as hypotension, tachycardia, or bradycardia, and corresponding symptoms (e.g., dizziness and headache, palpitation, and even syncope) occur in OI children after prolonged standing, during emotional stress, or in crowded or unventilated environments^2^2. Several mechanisms have been documented for pediatric OI, including autonomic nerve dysfunction, hypovolemia, and injured vascular endothelium, but the exact pathogenesis of OI remains unclear [12]. Children with OI have relatively higher plasma Hcy levels than normal children, suggesting high plasma Hcy might participate in the pathogenesis of OI [4, 5]. Hcy is an important metabolite of one-carbon metabolism, and elevated Hcy may be accompanied by a variety of metabolic changes. Therefore, to clarify the plasma metabolomic changes in OI children with high Hcy levels and investigate the potential underlying mechanism, we used UHPLC–Q-TOF MS analysis.

We found 105 significantly differential metabolites between the OI group and the control group. Several metabolites related to choline and glutamate changed significantly in the OI group. The upregulated metabolites included choline, PC(18:0/18:2(9*Z*,12*Z*)), PC(P-18:0/22:6(4*Z*,7*Z*,10*Z*,13*Z*,16*Z*,19*Z*)), histidine, isocitric acid, and DL-glutamic acid and its downstream metabolites. The downregulated metabolites included LPC(16:0), LPC(18:0), sphingomyelin (d18:1/18:0), betaine aldehyde, hydroxyproline, and GABA. Heatmaps revealed a common metabolic pattern with higher choline, PC(18:0/18:2(9*Z*,12*Z*)), and DL-glutamic acid, and lower sphingomyelin (d18:1/18:0), LPC(18:0), and LPC(16:0) in the subset of OI patients we called the special group compared with the common group and normal controls. Clinical indices analysis revealed that the max-uHR, ΔHR, and ΔHR/sHR × 100% of VVS patients were significantly higher in the special group than in the common group (*P <* 0.05). Choline, PC(18:0/18:2(9*Z*,12*Z*)), and DL-glutamic acid were positively correlated with ΔHR/sHR × 100% in VVS patients (*P <* 0.05).

Choline is an essential micronutrient for the human body, which is mainly derived from dietary intake or glycerophospholipid metabolism [13]. LPC(16:0) and LPC(18:0) are the upstream metabolites of choline in glycerophospholipid metabolism. In this study, choline was upregulated, whereas LPC(16:0) and LPC(18:0) were downregulated in the OI group, indicating that the elevated choline in OI children with high Hcy might be caused by increased dietary intake of it. Like folic acid, choline and betaine, can also be used as methyl group donors for one-carbon metabolism [14]. In mitochondria, choline dehydrogenase catalyzes the conversion of choline to betaine aldehyde, which is then transformed to betaine, thereby providing methyl groups for the remethylation of Hcy in one-carbon metabolism [14, 15]. More choline may be absorbed from the diet in the case of folate acid deficiency or utilization disorders [15–17]. Therefore, we suppose that OI children with high levels of Hcy might have folate acid deficiency or utilization disorders, resulting in increased choline intake and elevated plasma choline levels. We also found that betaine aldehyde was downregulated, whereas other downstream products of choline, such as PC(18:0/18:2(9*Z*,12*Z*)) and PC(P-18:0/22:6(4*Z*,7*Z*,10*Z*,13*Z*,16*Z*,19*Z*)), were upregulated in the OI group. These results suggested that OI children with high levels of Hcy might have difficulties in converting choline to betaine aldehyde, which affected one-carbon metabolism and increased Hcy levels. Further studies are necessary to confirm these speculations and investigate potential mechanisms.

A number of cardiovascular risk factors, including elevated SBP, increased BMI, insulin resistance, and decreased high-density lipoprotein cholesterol, have been linked to high choline levels and low betaine levels [18]. In Chinese adults with hypertension, Song et al. suggested that the serum choline concentration was positively correlated with all-cause mortality risk. The risk of all-cause death in hypertensive patients with serum choline level ≥ 4.00 µg/mL was 1.79 times higher than that in other hypertensive patients (*P* < 0.05) [16]. Prospective cohort studies of black people, white Americans, and Chinese adults have shown that high choline intake increased the mortality of individuals with cardiovascular diseases, especially diabetes and ischemic heart disease [19]. The underlying reasons for this risk remain unclear but may partly involve the increased generation of trimethylamine-*N*-oxide [18, 19]. Studies on choline and its upstream and downstream metabolites in OI are rare. Autonomic nerve dysfunction is a vital mechanism of OI, and the autonomic nuclei that control the autonomic nervous system are mostly located in the dorsal medulla [12, 20]. Using proton magnetic resonance spectroscopy, Wagoner et al. [21] found that OI children had higher levels of total choline in the dorsal medulla while in a supine position and that total choline was negatively correlated with the high-frequency α-index and sequence all. In our study, the plasma choline levels were also higher in the OI group than in the control group. Choline and its downstream product, PC(18:0/18:2(9*Z*,12*Z*)), were positively correlated with ΔHR/sHR × 100% in VVS patients. The high-frequency α-index, sequence all, and ΔHR/sHR × 100% are indices that reflect autonomic nerve function and the severity of OI [4, 21]. Therefore, the above studies indicate that high choline levels are associated with autonomic nerve dysfunction and might be involved in the pathogenesis of OI in children.

In the central nervous system (CNS), glutamate acts as the main excitatory neurotransmitter, while GABA acts as the main inhibitory neurotransmitter. The balance between glutamate and GABA is essential for maintaining the normal function of the nervous system. In experimental models, Hcy induced glutamatergic alterations in astrocytes, which was related to the decrease in the activity of Na^+^/K^+^-ATPase, an enzyme responsible for glutamate uptake [6]. Hcy also altered glutamate and GABA levels by activating the *N*-methyl-d-aspartate receptor in the CNS, whereas changes in glutamate and GABA levels differ between different regions of the CNS [22, 23]. In methylenetetrahydrofolate reductase–deficient mice, Jadavji et al. [23] reported that glutamate was elevated in the amygdala, GABA dropped in the thalamus, and both were decreased in the hippocampus. The essential enzyme responsible for converting glutamic acid into GABA is glutamic acid decarboxylase (GAD). After 15 days of l-methionine feeding, there was an increase in *S*-adenosyl-homocysteine and a decrease in GAD_67_ mRNA in mouse brain tissues, which might be caused by CpG island hypermethylation in the *GAD*_*67*_ promoter region [24]. Afterward, the downregulation of GAD_67_ mRNA expression affected the glutamate and GABA levels. The aforementioned studies suggest that high Hcy can cause an imbalance between glutamate and GABA in the CNS.

The glutamate–GABA imbalance has been linked to various neurological disorders in children, such as migraine, depression, and attention deficit and hyperactivity disorder (ADHD) [25–27]. Using magnetic resonance spectroscopy (MRS), Bell et al. found that the glutamate–GABA imbalance could occur in the early stage of pediatric migraine. The glutamate concentration in the thalamus and the GABA/Glx (a combination of glutamate and glutamine) ratio in the sensorimotor cortex increased with the duration of migraine [25]. Proton magnetic resonance spectroscopy showed that young patients with depression had notably lower GABA levels in the anterior cingulate cortex compared to healthy controls [26]. The levels of amino acid neurotransmitters in serum are closely related to those in the brain and cerebrospinal fluid. Miniksar et al. [27] found that individuals with ADHD had elevated serum levels of Hcy, glutamate, and GABA levels, which could be used as predictor biomarkers for ADHD.

OI children are often concomitant with neurological disorders, which can be explained by potential shared mechanisms [1, 28, 29]. Wang et al. [30] reported that among 85 VVS children with comorbidities, 14.1% (12/85) had psychological disorders and 3.6% (3/85) had migraine. In individuals with POTS, the proportion of migraine was higher: 50.1% (95% CI 9.9–90.3) in adolescents and 31.2% (95% CI 5.4–57.0) in adult patients [31]. As observed in neurological disorders, plasma DL-glutamic acid was higher and plasma GABA was lower in our OI group than our control group, indicating that the glutamate–GABA imbalance also occurred in OI children with high Hcy. DL-glutamic acid was positively correlated with ΔHR/sHR × 100% in VVS patients (*P <* 0.05), suggesting that glutamate–GABA imbalance would result in negative effects on OI children. Glutamatergic excitotoxicity or other mechanisms might be involved in this process, requiring further studies [32].

The current research has shortcomings. For instance, the relatively small sample size might have resulted in bias, and OI patients with a normal level of plasma Hcy were not included, which made it unfeasible to investigate the plasma metabolomic changes in OI children with different levels of Hcy. The causes of high plasma Hcy in children with OI, such as deficiencies in folic acid and B-group vitamins, declines in Hcy-metabolizing enzyme function, or mutations in gene-encoding enzymes, were not investigated.

## Conclusions

Despite these shortcomings, our research is the first to explore the plasma metabolomic profile in OI children with high levels of plasma Hcy. We found that choline-related metabolites and glutamate-related metabolites changed significantly in OI children with high Hcy and that these changes were associated with the severity of illness, providing a new light on the pathogenesis of OI. More studies are necessary to verify our results and explore the underlying mechanisms. It is also worth exploring whether these metabolic changes in OI children with high Hcy are reversed by reducing their Hcy levels.

### Electronic supplementary material

Below is the link to the electronic supplementary material.

**Supplementary Material: Table S1.** Significantly differential metabolites

**Supplementary Material: Figure S1.** Arginine and proline metabolism. Red points and blue points are upregulated and downregulated metabolites, respectively

**Supplementary Material: Figure S2.** Biosynthesis of amino acids

**Supplementary Material: Figure S3.** Heatmap of negative ion mode. Each row in the y-axis represents a significantly differential metabolite and each column in the x-axis represents a set of samples. Red represents significant upregulation of the metabolite, and blue represents significant downregulation of the metabolite. Color depth represents the degree of upregulation and downregulation. The metabolites with similar expression patterns are clustered in the same cluster on the left. C: Control group. OI: OI group

**Supplementary Material: Figure S4.** Correlation-based network of negative ion mode. Each point represents a significantly differential metabolite. The red point is what we focus on. The sizes of the points increase with degree. Red and blue lines between the metabolites indicate positive and negative correlations, respectively (Spearman correlation coefficient |r| > 0.8 and P < 0.05). The thickness of lines represents absolute correlation coefficient. The line becomes thicker as the absolute correlation coefficient increases

**Supplementary Material: Table S2.** Baseline characteristics and hemodynamic indices of POTS patients

**Supplementary Material: Figure S5.** Relationship between metabolites and △HR/sHR × 100% in POTS patients. △HR/sHR × 100%, rate of change in heart rate from the supine to the upright position; PC(18:0/18:2(9Z,12Z)), 1-stearoyl-2-linoleoyl-sn-glycero-3-phosphocholine

**Supplementary Material:** Graphical abstract

## Data Availability

The original contributions presented in the study are included in the supplementary material. Further inquiries can be directed to the corresponding authors.

## Abbreviations

ADHD: attention deficit and hyperactivity disorder
BHUTT: basic head-up tilt test
BMI: body mass index
BP: blood pressure
CNS: central nervous system
ECG: electrocardiogram
FC: Fold change
GABA: gamma-aminobutyric acid
GAD: glutamic acid decarboxylase
HCA: hierarchical clustering analysis
Hcy: homocysteine
HR: heart rate
HUTT: head-up tilt test
KEGG: Kyoto Encyclopedia of Genes and Genomes
LPC (16:0): 1-palmitoyl-sn-glycero-3-phosphocholine
LPC (18:0): 1-stearoyl-sn-glycerol 3-phosphocholine
MRS: magnetic resonance spectroscopy
OI: Orthostatic intolerance
OPLS-DA: orthogonal partial least-squares discriminant analysis
PC(18:0/18:2(9*Z*,12*Z*)): 1-stearoyl-2-linoleoyl-*sn*-glycero-3-phosphocholine
PC(P-18:0/22:6(4*Z*,7*Z*,10*Z*,13*Z*,16*Z*,19*Z*)): 1-(1*Z*-octadecenyl)-2-(4*Z*,7*Z*,10*Z*,13*Z*,16*Z*,19*Z*-docosahexaenoyl)-*sn*-glycero-3-phosphocholine
PCA: Pareto-scaled principal component analysis
POTS: postural orthostatic tachycardia syndrome
sDBP: mean supine diastolic blood pressure
sHR: mean supine heart rate
SNHUTT: sublingual nitroglycerin-provoked head-up tilt test
sSBP: mean supine systolic blood pressure
UHPLC-Q-TOF MS: ultra-high-pressure liquid chromatography and quadrupole-time-of-flight mass spectrometry
VIP: variable importance in projection
VVS: vasovagal syncope
VVS-CI: vasovagal syncope cardioinhibitory type
VVS-M: vasovagal syncope mixed type
VVS-VI: vasovagal syncope vasoinhibitory type
