# Supplementary figures and images for: Plasma metabolomic profile in orthostatic intolerance children with high levels of plasma homocysteine
Source: Ital J Pediatr. 2024 Mar 14;50:52. doi: 10.1186/s13052-024-01601-4 (PMC10941598; doi:10.1186/s13052-024-01601-4)

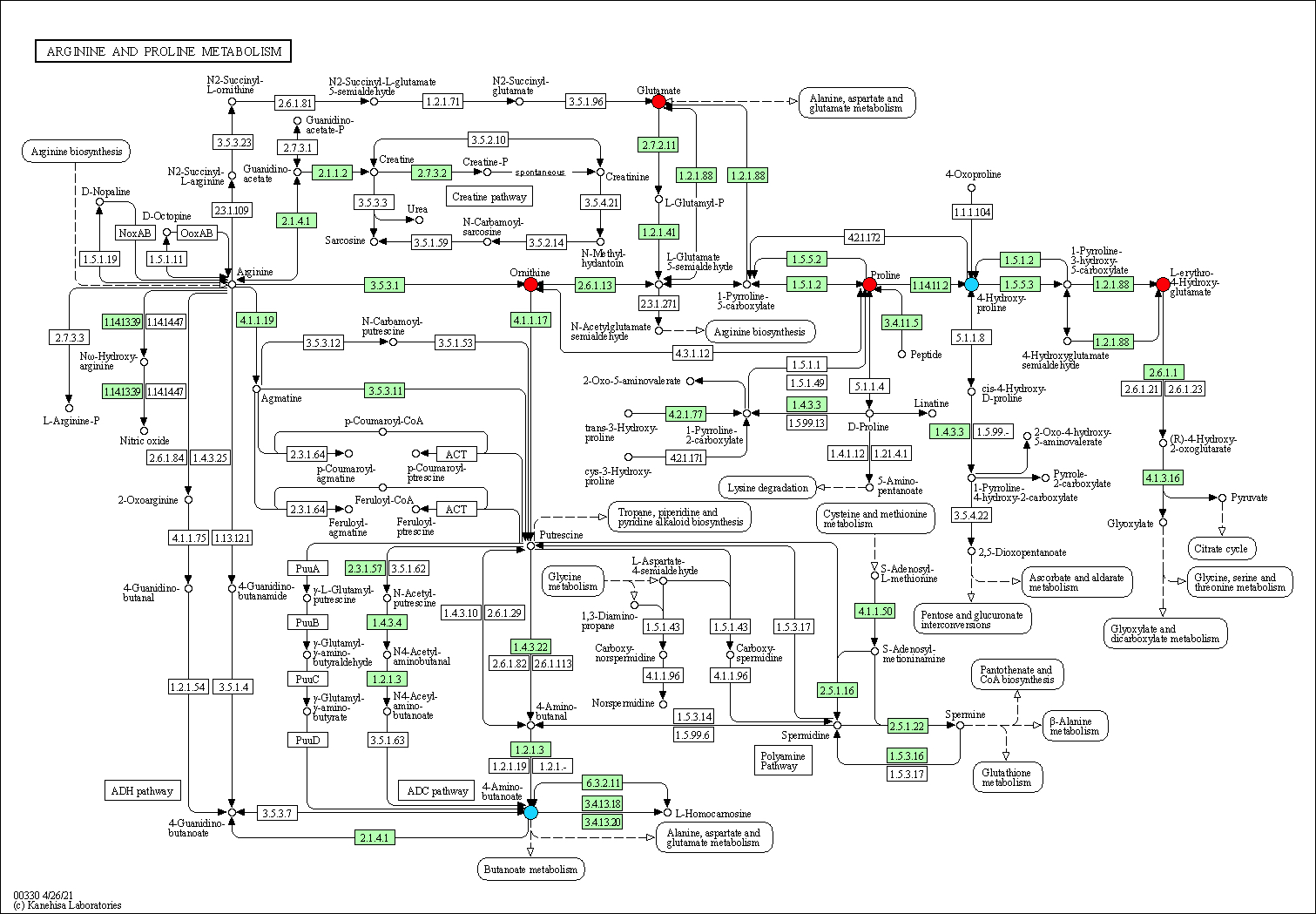

Supplement: Supplementary file 2 — Supplementary Material: Figure S1. Arginine and proline metabolism. Red points and blue points are upregulated and downregulated metabolites, respectively [file 13052_2024_1601_MOESM2_ESM.jpg]

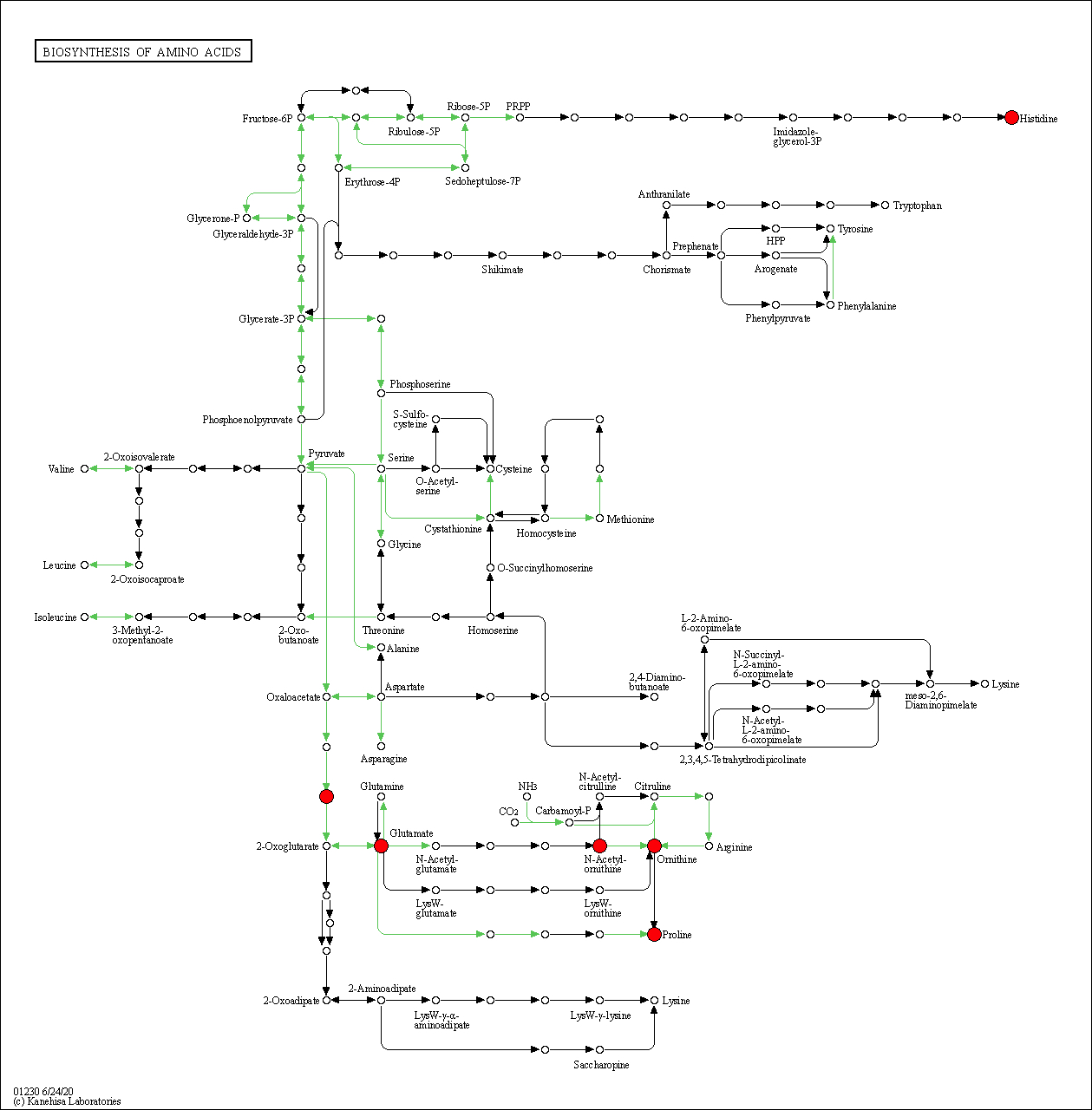

Supplement: Supplementary file 3 — Supplementary Material: Figure S2. Biosynthesis of amino acids [file 13052_2024_1601_MOESM3_ESM.jpg]

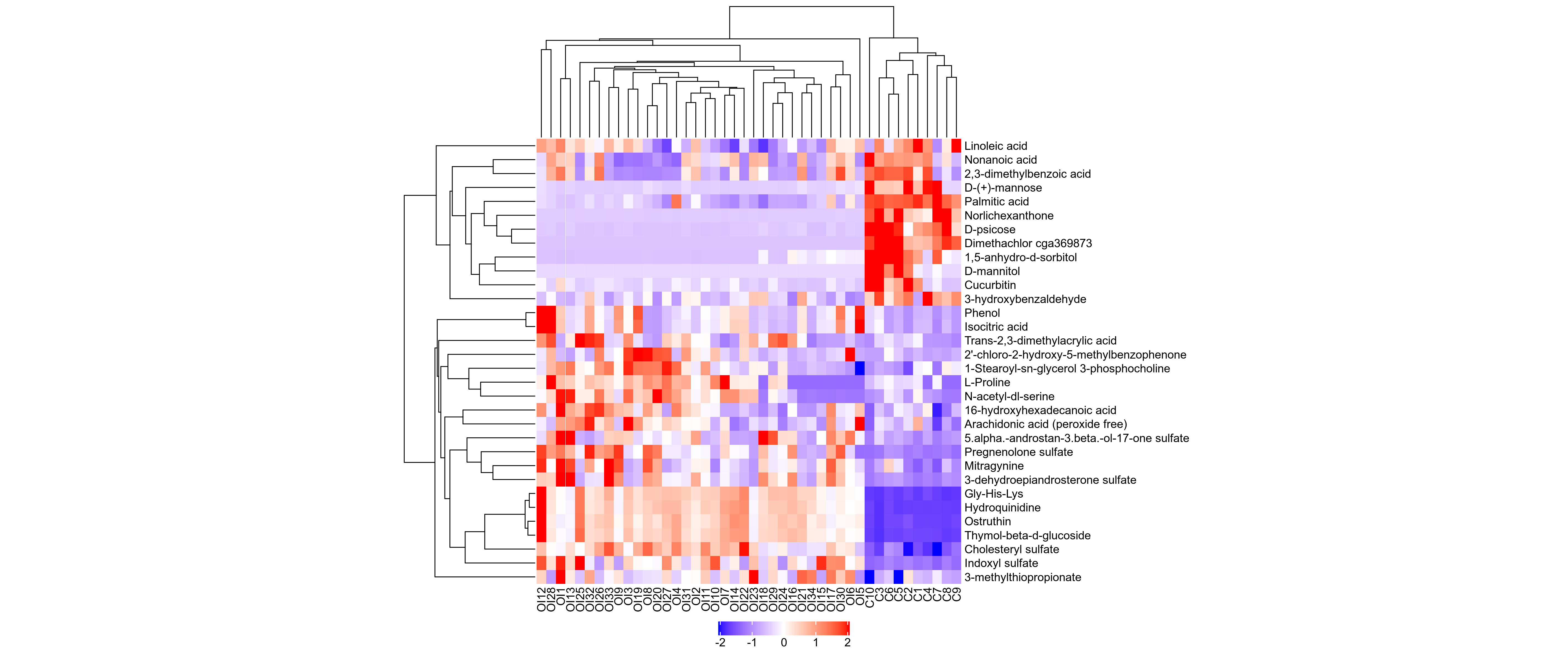

Supplement: Supplementary file 4 — Supplementary Material: Figure S3. Heatmap of negative ion mode. Each row in the y-axis represents a significantly differential metabolite and each column in the x-axis represents a set of samples. Red represents significant upregulation of the metabolite, and blue represents significant downregulation of the metabolite. Color depth represents the degree of upregulation and downregulation. The metabolites with similar expression patterns are clustered in the same cluster on the left. C: Control group. OI: OI group [file 13052_2024_1601_MOESM4_ESM.jpg]

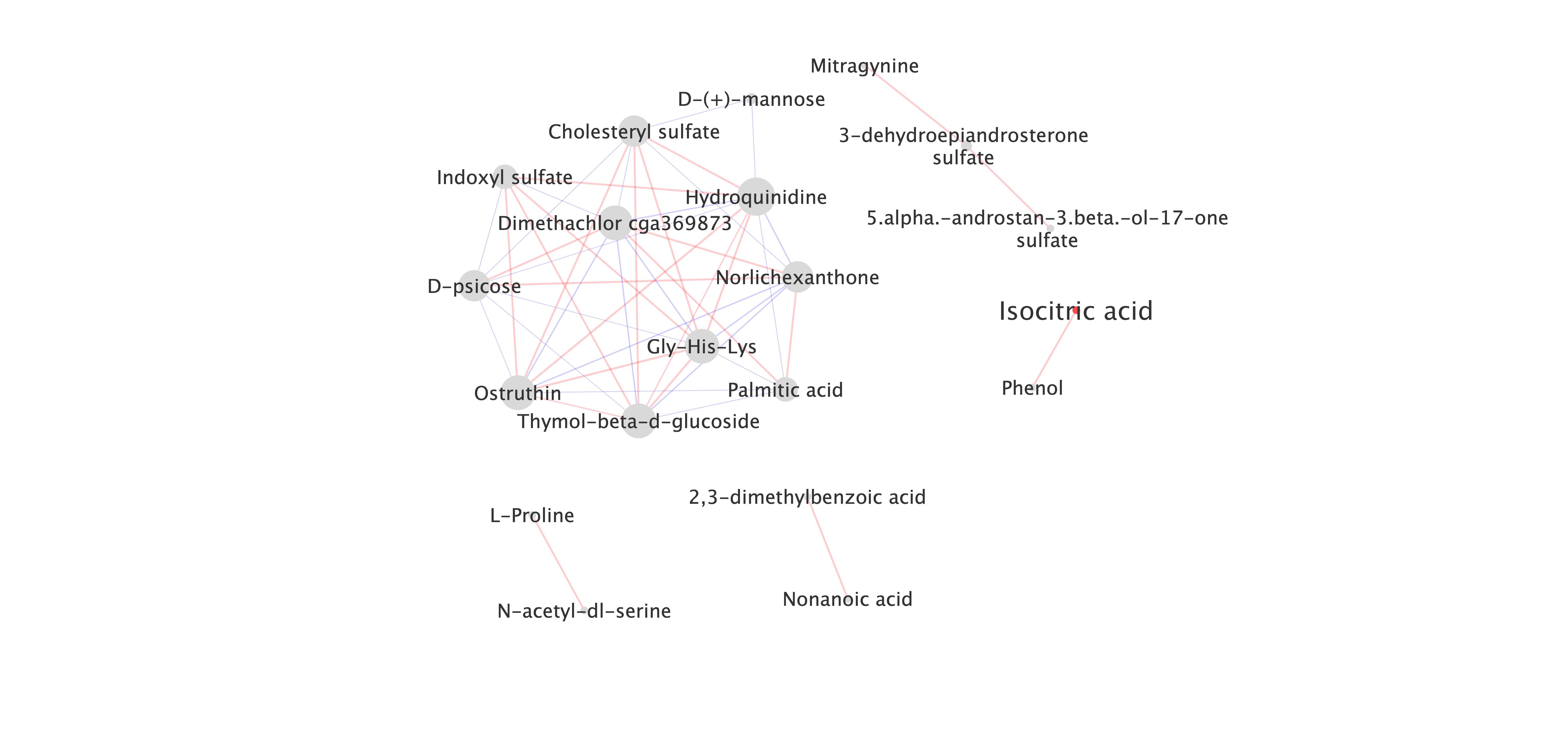

Supplement: Supplementary file 5 — Supplementary Material: Figure S4. Correlation-based network of negative ion mode. Each point represents a significantly differential metabolite. The red point is what we focus on. The sizes of the points increase with degree. Red and blue lines between the metabolites indicate positive and negative correlations, respectively (Spearman correlation coefficient |r| > 0.8 and P < 0.05). The thickness of lines represents absolute correlation coefficient. The line becomes thicker as the absolute correlation coefficient increases [file 13052_2024_1601_MOESM5_ESM.jpeg]

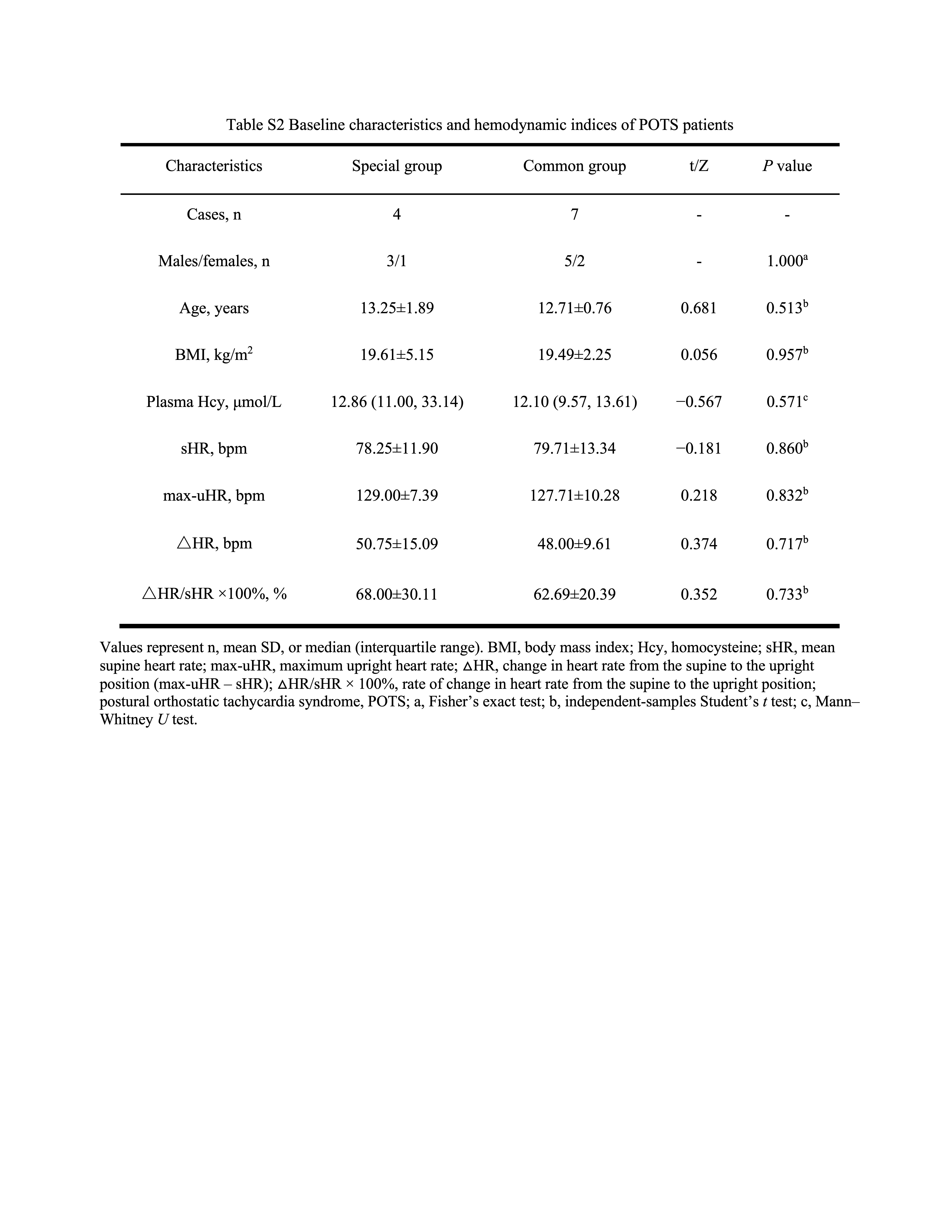

Supplement: Supplementary file 6 — Supplementary Material: Table S2. Baseline characteristics and hemodynamic indices of POTS patients [file 13052_2024_1601_MOESM6_ESM.jpg]

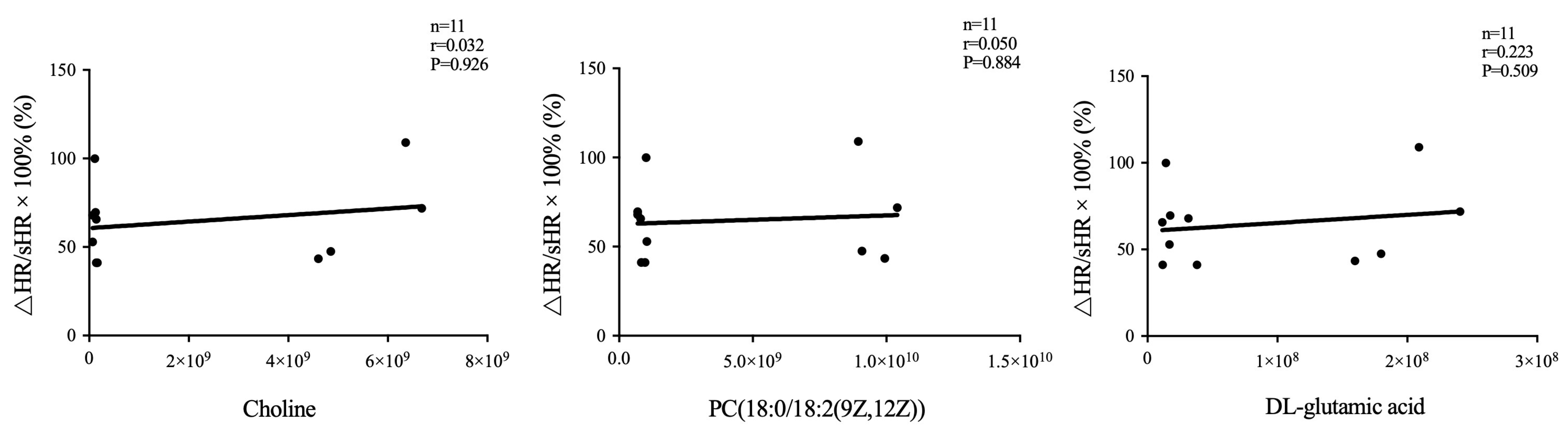

Supplement: Supplementary file 7 — Supplementary Material: Figure S5. Relationship between metabolites and △HR/sHR × 100% in POTS patients. △HR/sHR × 100%, rate of change in heart rate from the supine to the upright position; PC(18:0/18:2(9Z,12Z)), 1-stearoyl-2-linoleoyl-sn-glycero-3-phosphocholine [file 13052_2024_1601_MOESM7_ESM.png]

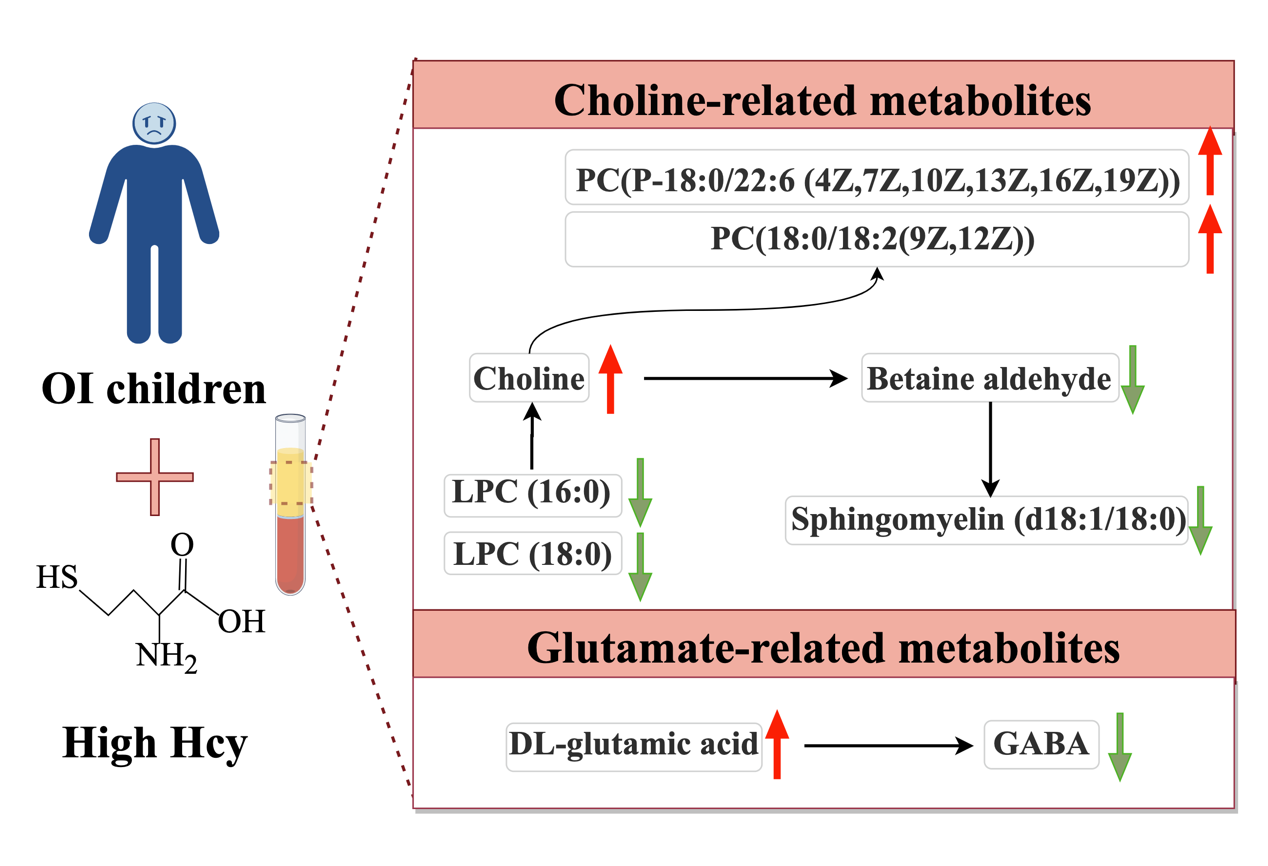

Supplement: Supplementary file 8 — Supplementary Material: Graphical abstract [file 13052_2024_1601_MOESM8_ESM.docx]
